# Supplementary material for: COVID-19 Vaccination in Pregnancy: Pilot Study of Plasma MicroRNAs Associated with Inflammatory Cytokines after COVID-19 mRNA Vaccination
Source: Vaccines (Basel). 2024 Jun 14;12(6):658. doi: 10.3390/vaccines12060658 (PMC11209245; doi:10.3390/vaccines12060658)
Supplement: Supplementary file 1 [file vaccines-12-00658-s001.zip › vaccines-3017192-supplementary.pdf]

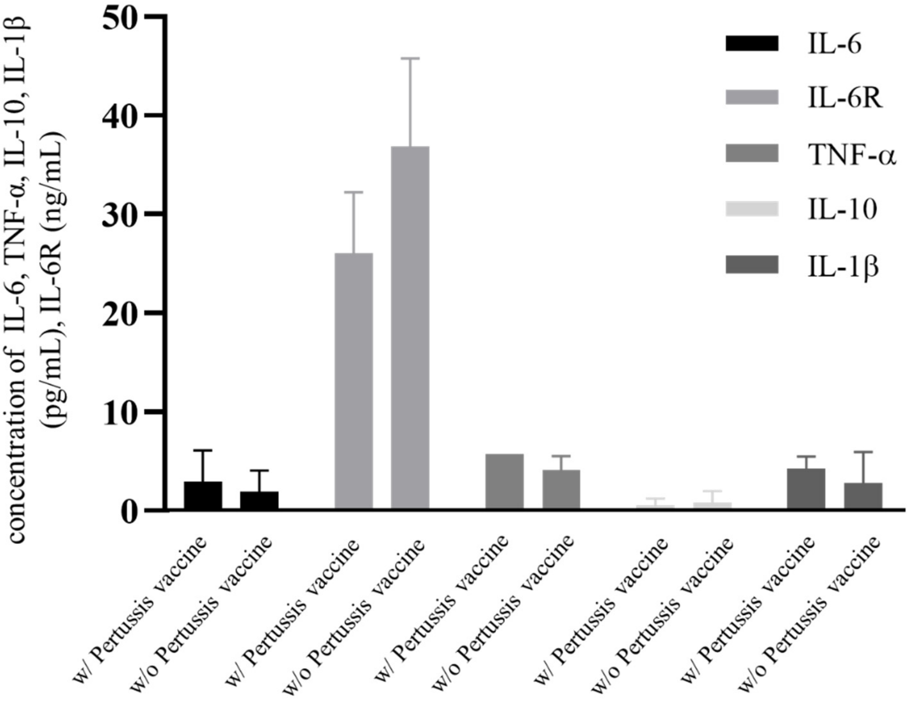

Supplementary Figure S1. IL-6, IL-6R, TNF-α, IL-10 & IL-1β concentration (Week range = 1–8 weeks; one dose: w/Pertussis vaccine, n = 21; w/o Pertussis vaccine, n = 21).

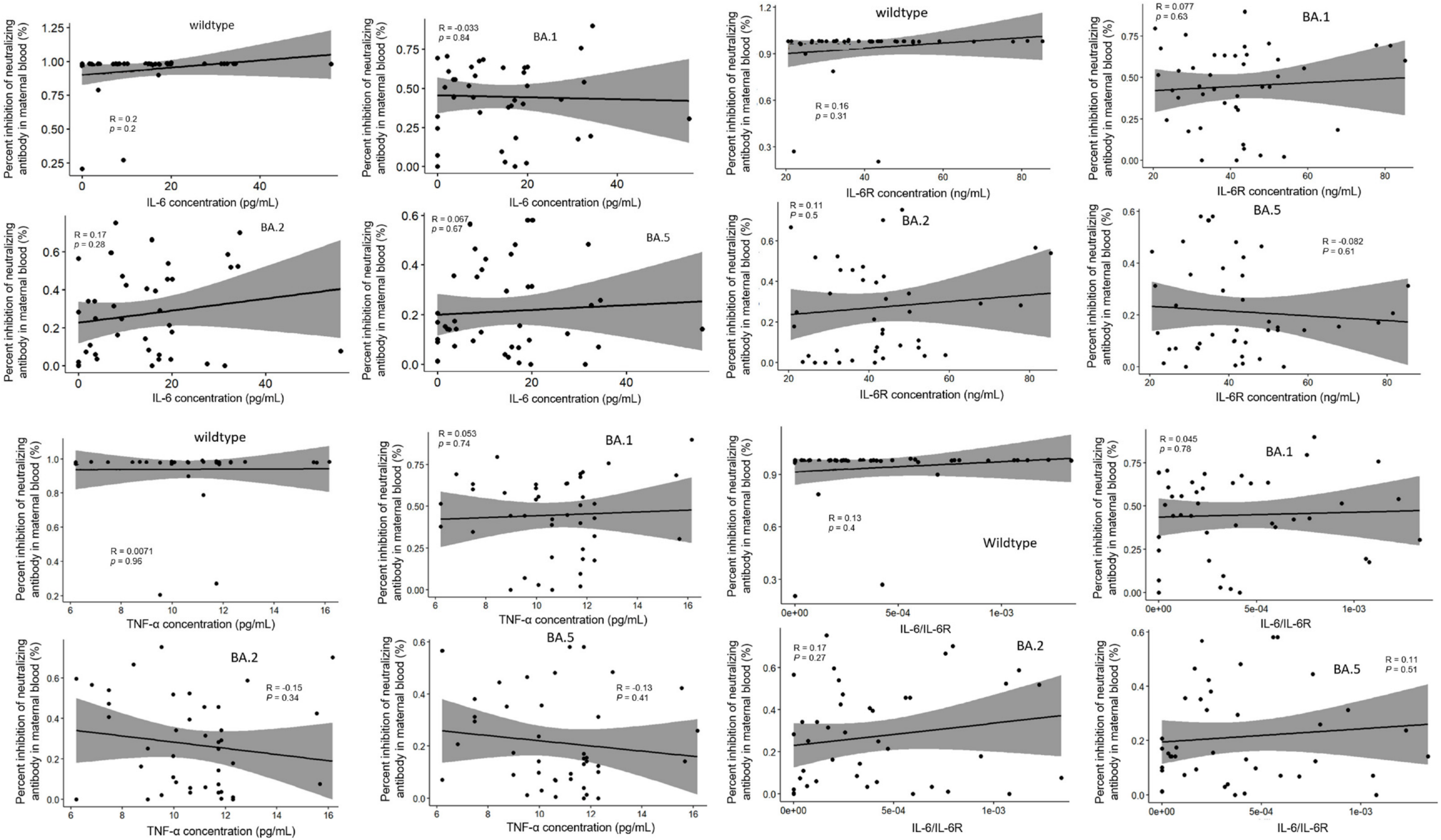
wildtype
R = 0.2
p = 0.2
IL-6 concentration (pg/mL)
Percent inhibition of neutralizing antibody in maternal blood (%)
R = -0.033
p = 0.84
BA.1
IL-6 concentration (pg/mL)
wildtype
R = 0.16
p = 0.31
IL-6R concentration (ng/mL)
R = 0.077
p = 0.63
BA.1
IL-6R concentration (ng/mL)
R = 0.17
p = 0.28
BA.2
IL-6 concentration (pg/mL)
R = 0.067
p = 0.67
BA.5
IL-6 concentration (pg/mL)
R = 0.11
P = 0.5
BA.2
IL-6R concentration (ng/mL)
BA.5
R = -0.082
P = 0.61
IL-6R concentration (ng/mL)
wildtype
R = 0.0071
p = 0.96
TNF-α concentration (pg/mL)
R = 0.053
p = 0.74
BA.1
TNF-α concentration (pg/mL)
R = 0.13
p = 0.4
Wildtype
IL-6/IL-6R
R = 0.045
p = 0.78
BA.1
IL-6/IL-6R
BA.2
R = -0.15
P = 0.34
TNF-α concentration (pg/mL)
BA.5
R = -0.13
P = 0.41
TNF-α concentration (pg/mL)
R = 0.17
P = 0.27
BA.2
IL-6/IL-6R
BA.5
R = 0.11
P = 0.51
IL-6/IL-6R

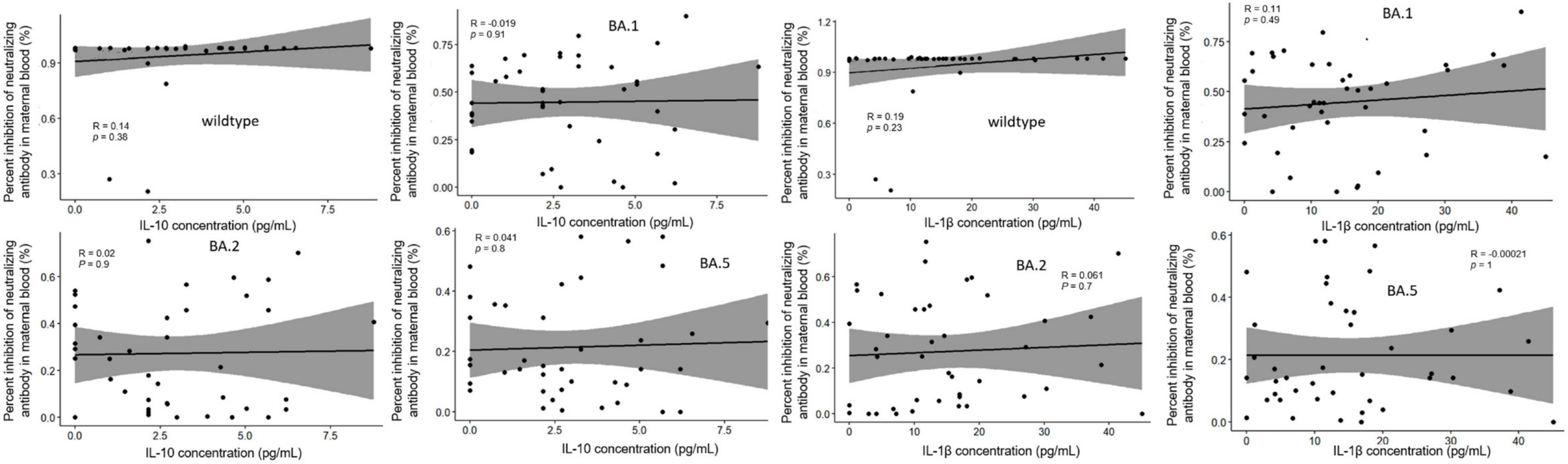

Supplementary Figure S2. Correlation between the neutralizing antibody inhibition rate of different variants and IL-6, IL-6R, TNF-α, IL-10, IL-1β concentration and IL-6/IL-6R in pregnant women who received three doses of mRNA vaccine (n = 42).
